# A Pan-Cancer Analysis of CD161, a Potential New Immune Checkpoint

**DOI:** 10.3389/fimmu.2021.688215

**Published:** 2021-07-09

**Authors:** Xiaohan Zhou, Jun Du, Chengdong Liu, Hanyi Zeng, Yuting Chen, Li Liu, Dehua Wu

**Affiliations:** ^1^ Department of Radiation Oncology, Nanfang Hospital, Southern Medical University, Guangzhou, China; ^2^ National Cancer Center/National Clinical Research Center for Cancer/Cancer Hospital, Chinese Academy of Medical Sciences and Peking Union Medical College, Beijing, China; ^3^ Department of Infectious Diseases, Nanfang Hospital, Southern Medical University, Guangzhou, China

**Keywords:** TCGA, immune microenvironment, CD161, immune checkpoints, immunotherapy

## Abstract

**Background:**

CD161, encoded by killer cell lectin-like receptor B1 gene, is a newly reported candidate inhibitor of tumour-infiltrating T cells. Antibody-mediated CD161 blockade enhances T cell-mediated killing of cancer cells *in vitro* and *in vivo* in several tumour types. We evaluated the role of CD161 using The Cancer Genome Atlas (TCGA) Pan-Cancer Data.

**Methods:**

CD161 expression was analysed using RNAseq data from TCGA and the Genotype-Tissue Expression (GTEx) database. HPA, GeneCards, and String database were used to explore the protein information of CD161. The prognostic value of CD161 was analysed using clinical survival data from the TCGA. Enrichment analysis of CD161 was conducted using the R package “clusterProfiler”. We downloaded the immune cell infiltration score of TCGA samples from published articles and online databases and performed a correlation analysis between immune cell infiltration levels and CD161 expression. We further assessed the association between CD161 and immune checkpoints, immune activating genes, immunosuppressive genes, chemokines, and chemokine receptors.

**Findings:**

CD161 was differentially expressed and predicted better survival status in most tumour types in TCGA. In addition, CD161 expression was significantly associated with immunoregulatory interactions between lymphoid and non-lymphoid cells. CD161 expression was closely correlated with T cell infiltration, immune checkpoints, immune activating genes, immunosuppressive genes, chemokines, and chemokine receptors.

**Interpretation:**

Our results suggest that CD161 is a potential cancer biomarker. CD161 might synergize with other immune checkpoints to regulate the immune microenvironment, which could be applied in the development of new-targeted drugs for immunotherapy.

**Funding:**

This work was supported by the National Nature Science Foundation of China (grant numbers 81773008, 81672756, 81872399, 81972897), the Guangdong Province Universities and Colleges Pearl River Scholar Funded Scheme (2015), the Natural Science Foundation of Guangdong Province (grant number 2017A030311023), the Local Innovative and Research Teams Project of Guangdong Pearl River Talents Program: 2017BT01S131 and the Guangzhou Technology Project (grant number 201804010044), National Key R&D Program of China (Grant Nos. 2020YFC2006400), Key-Area Research and Development Program of Guangdong Province (2019B020227004).

## Introduction

The accumulation of a variety of genetic alterations leading to the expression of different new antigens on the surfaces of cancer cells is an important characteristic of tumours (1). However, cancer cells have also developed complex ways to escape from attacks by the immune system, and these ways are a major obstacle to successful cancer immunotherapy (2, 3). To date, many immune escape mechanisms have been identified, including the expression of endogenous “immune checkpoints” that usually alleviate the immune response after antigen activation. These phenomena have led to the development of immune checkpoint inhibitors as anticancer drugs, including anti-programmed cell death protein 1 (PD-1), anti-cytotoxic T-lymphocyte associated protein 4 (CTLA4), and anti-lymphocyte activation gene 3 (LAG3).

CD161 is expressed on natural killer cells, subsets of CD4^+^ and CD8^+^ T cells (4). A recent study using single-cell RNA sequencing of tumour-infiltrating T cells discovered that CD161 acts as a potential inhibitory receptor in liver cancer and glioma. C-Type lectin domain family 2 member D, the ligand of CD161, is expressed on the cell membranes of malignant tumour cells, forming a ligand-receptor pathway for immunotherapy (5, 6). Previous research has proven that the expression of CD161 in lung cancer is associated with better clinical outcomes (7). Another study proved that CD8+PD-1+CD161+ T cell subsets have stronger cytotoxicity and proliferative capacity in hepatocellular carcinoma (8). In addition, blocking he interaction could enhance NK cell-mediated lysis in triple-negative breast cancer (9). These results indicated an essential role of CD161 in tumour immunomodulation. However, there have been no comprehensive pan-cancer studies on CD161.

In this study, we evaluated the expression of CD161 and its association with the prognosis of patients with cancer. We further examined the association between CD161 and the immune cell infiltration score, immune checkpoints, immune activating genes, immunosuppressive genes, chemokines, and chemokine receptors. Our results provide novel insights into the functional role of CD161 in pan-cancer, highlighting a potential mechanism whereby CD161 influences the tumour microenvironment, as well as cancer immunotherapy.

## Methods

### Data Collection

RNA expression and clinical data of The Cancer Genome Atlas (TCGA) and Genotype-Tissue Expression (GTEx) were downloaded from the UCSC Xena database (https://xenabrowser.net/datapages/). The DNA copy number and methylation data were downloaded from the cBioPortal database (https://www.cbioportal.org/).

### Protein Level Analysis

The Human Protein Atlas (HPA: https://www.proteinatlas.org/) database was used to explore the protein level of CD161 in human tumor and normal tissues. String (https://string-db.org/) database was used to construct the protein-protein interaction network (PPI) of CD161. GeneCards (https://www.genecards.org/) was used to visualise the subcellular locations of CD161.

### Immune Cell Infiltration

We downloaded and analyzed the immune cell infiltration score of TCGA from the ImmuCellAI database (http://bioinfo.life.hust.edu.cn/web/ImmuCellAI/), TIMER2 database (http://timer.cistrome.org/), and a previously published study (10, 11). For each TCGA tumour type, patients were divided into two groups (high and low CD161 expression based on the median CD161 expression level) to compare the extent of immune cell infiltration.

### Prognostic Analysis

Kaplan-Meier analysis was performed to evaluate the overall survival (OS) of patients from TCGA cohort. Univariate Cox regression analyses were conducted to assess the significance of CD161 in predicting OS, disease-specific survival (DSS), the disease-free interval (DFI), and the progression-free interval (PFI) in pan-cancer.

### Gene Set Enrichment Analyses

Correlation analyses of CD161 with all genes was performed using TCGA data. Pearson’s correlation coefficients were calculated. Genes correlated with CD161 (p < 0.05) were selected for gene set enrichment analysis (GSEA). GSEA was performed using the R package “clusterProfiler” with the following parameters: nPerm = 1000, minGSSize = 10, maxGSSize = 1000, and p-value-Cutoff = 0.05 (12). Gene sets from Reactome pathway database were selected for GSEA.

### Statistical Analyses

Data are presented as means ± standard error (SD). Differences between groups were analyzed using a Student’s t-test. Statistical analyses were performed using R 3.6.2. P < 0.05 (two-tailed) was considered statistically significant.

## Results

### CD161 Expression Analysis in Pan-Cancer

**Figure 1:**
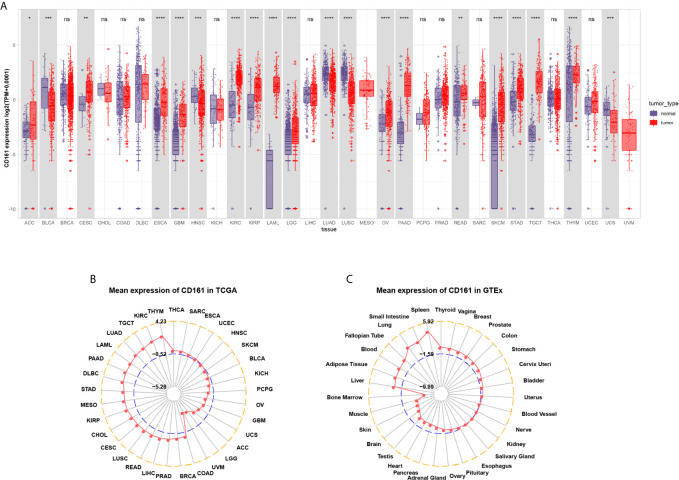
Pan-cancer CD161 expression. **(A)**, Pan-cancer expression of CD161 between tumor tissues from TCGA database and normal tissues from TCGA and GTEx database. **(B)**, CD161 expression in tumor tissues from TCGA database. The location of the dot represents the mean value of CD161 expression. **(C)**, CD161 expression in normal tissues from GTEx database. The location of the dot represents the mean value of CD161 expression. *p < 0.05; **p < 0.01; ***p < 0.001 and ****p < 0.0001; ns, not significant.

We first evaluated CD161 expression in TCGA pan-cancer. The results revealed that high CD161 expression was observed in 14 tumours: ACC, CESC, ESCA, GBM, KIRC, KIRP, LAML, LGG, OV, PAAD, READ, SKCM, STAD, and TGCT. In comparison, low CD161 expression was observed in four tumours: BLCA, HNSC, LUAD, and LUSC (**Figure 1A**). In addition, for tumour tissues in TCGA, CD161 expression was the highest in THYM, KIRC, and LUAD (**Figure 1B**). For human normal tissues in GTEx, CD161 expression was the highest in the spleen, small intestine, and lung (**Figure 1C**).

**Figure 2:**
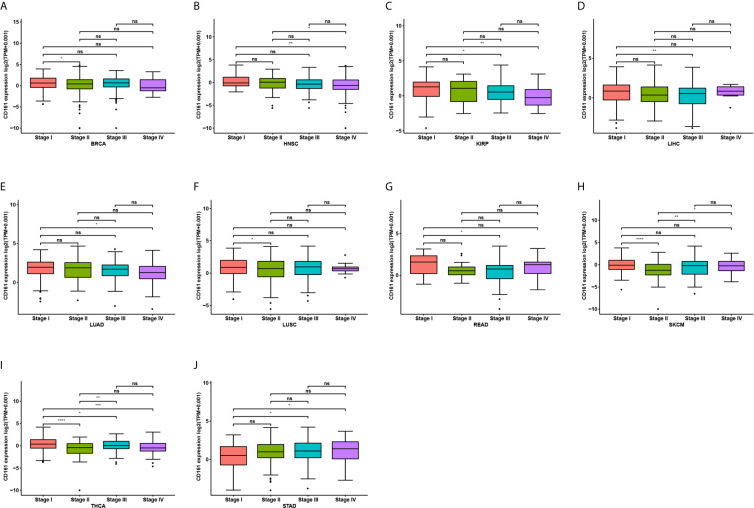
Pan-cancer CD161 expression in different WHO stages. **(A–J)**, Pan-cancer differential expression of CD161 in WHO stages in indicated tumor types from TCGA database. *p < 0.05; **p < 0.01; ***p < 0.001 and ****p < 0.0001; ns, not significant.

We further assessed CD161 expression in different World Health Organization cancer stages and found that it was lower in higher stages in most tumours, including BRCA, HNSC, KIRP, LIHC, LUAD, LUSC, READ, SKCM, and THCA (**Figures 2A–I**). In contrast, higher CD161 expression in higher stages was observed only in STAD (**Figure 2J**).

**Figure 3:**
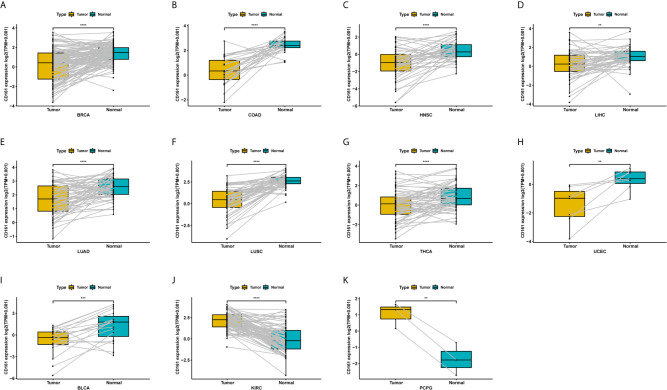
Pan-cancer paired CD161 expression. **(A–K)**, Pan-cancer differential expression of CD161 in paired tumor and adjacent normal tissues in indicated tumor types from TCGA database. **p < 0.01; ***p < 0.001 and ****p < 0.0001.

For paired tumour and normal tissues in TCGA pan-cancer, CD161 was expressed at low levels in BRCA, COAD, HNSC, LIHC, LUAD, LUSC, THCA, UCEC, and BLCA (**Figures 3A–I**), while it was highly expressed in KIRC and PCPG (**Figures 3J, K**).

### Genetic Alterations of CD161

Genetic and epigenetic alterations induce changes in gene expression. We explored genetic alterations of CD161 using cBioPortal and observed that patients with uterine carcinosarcoma or testicular germ cell tumours possessed a high frequency of gene alterations (**Supplementary Figure 1A**). Copy number values were positively correlated with CD161 expression only in KIRP (**Supplementary Figure 1B**). In addition, the methylation level of the CD161 promoter was negatively correlated with CD161 expression in LIRC, LGG, SKCM, LUAD, TGCT, and THYM (**Supplementary Figure 1C**).

### Protein Level of CD161

Above, we have analysed the mutation, copy number, methylation and mRNA expression of CD161. We continue to explore the protein level of CD161. Using HPA database, we found that the protein level of CD161 was highest in testis cancer, while lowest in lymphoma (**Supplementary Figure 2A**). For human normal tissues, CD161 protein level was high in tissues of kidney, epididymis, prostate, heart muscle, spleen, and bone marrow (**Supplementary Figure 2B**). As a potential immune checkpoint, location on cell membrane is necessary. We explored the subcellular location of CD161 using GeneCards database. We observed that the protein location of CD161 was mainly on the plasma membrane (**Supplementary Figure 2C**). In addition, we constructed the PPI network and found that CD161 (KLRB1) was closely associated with CLEC2D, CLEC2A, GZMA, GZMB, GZMK, KLRC4-KLRK1, CD2, CMKBR6, RORC, and GPR29 proteins (**Supplementary Figure 2D**).

### Prognostic Significance of CD161

**Figure 4:**
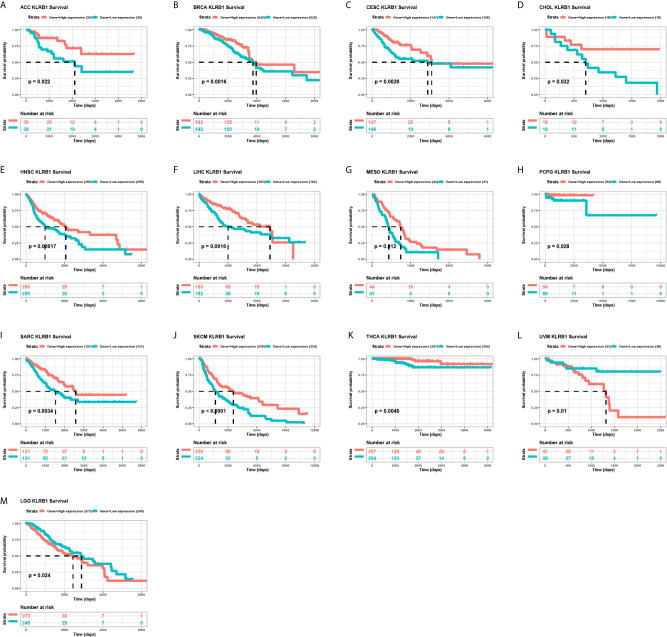
Kaplan-Meier overall survival of CD161. **(A–M)**, Pan-cancer Kaplan-Meier overall survival of CD161 in indicated tumor types from TCGA database. The median value of CD161 in each tumor was taken as the cut-off value.

**Figure 5:**
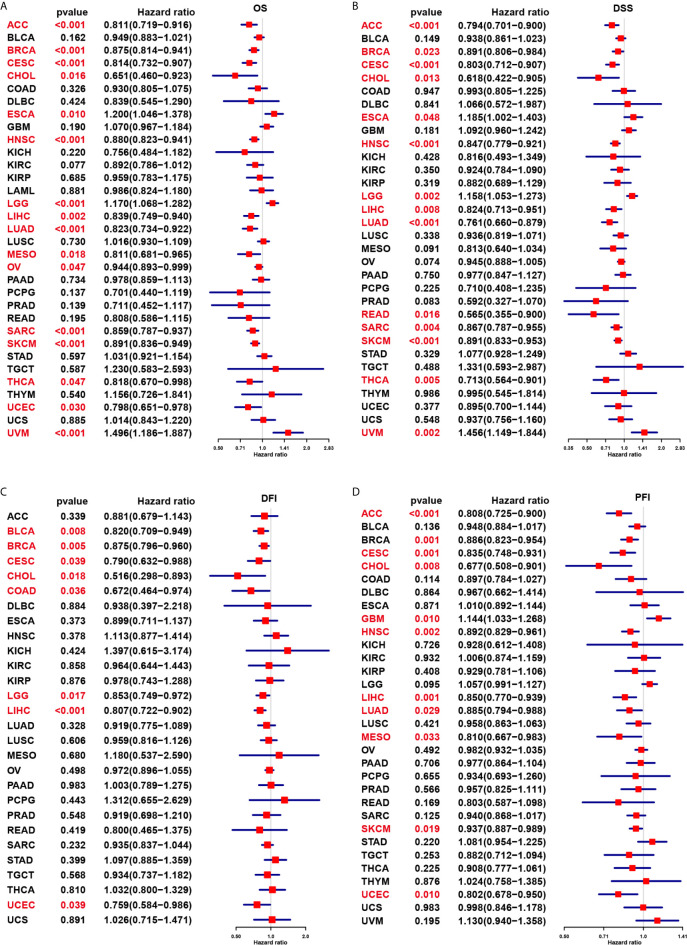
Univariate Cox regression analysis of CD161. **(A)**, Forest map shows the univariate cox regression results of CD161 for OS in TCGA pan-cancer. **(B)**, Forest map shows the univariate cox regression results of CD161 for DSS in TCGA pan-cancer. **(C)**, Forest map shows the univariate cox regression results of CD161 for DFI in TCGA pan-cancer. **(D)**, Forest map shows the univariate cox regression results of CD161 for PFI in TCGA pan-cancer. Red colors represent significant results.

We further evaluated the prognostic significance of CD161 in patients with cancer. The results of Kaplan-Meier OS analysis indicated that CD161 is a protective factor for patients with ACC, BRCA, CESC, CHOL, HNSC, LIHC, MESO, PRAD, PCPG, SARC, SKCM, and THCA (**Figures 4A–K**) and a risk factor for patients with UVM and LGG (**Figures 4L, M**). For the results of univariate Cox regression analyses, the OS results revealed that CD161 acts as a protective factor for patients with ACC, BRCA, CESC, CHOL, HNSC, LIHC, LUAD, MESO, OV, SARC, SKCM, THCA, and UCEC and a risk factor for patients with ESCA, LGG, and UVM (**Figure 5A**). The DSS analysis revealed that CD161 acts as a protective factor for patients with ACC, BRCA, CESC, CHOL, HNSC, LIHC, LUAD, READ, SARC, SKCM, and THCA and a risk factor for patients with ESCA, LGG, and UVM (**Figure 5B**). The DFI analysis revealed that CD161 acts as a protective factor for patients with BLCA, BRCA, CESC, CHOL, COAD, LGG, LIHC, and UCEC (**Figure 5C**). Finally, the PFI analysis showed that CD161 acts as a protective factor for patients with ACC, CESC, CHOL, HNSC, LIHC, LUAD, MESO, SKCM, and UCEC and a risk factor for patients with GBM (**Figure 5D**).

### GSEA of CD161

**Figure 6:**
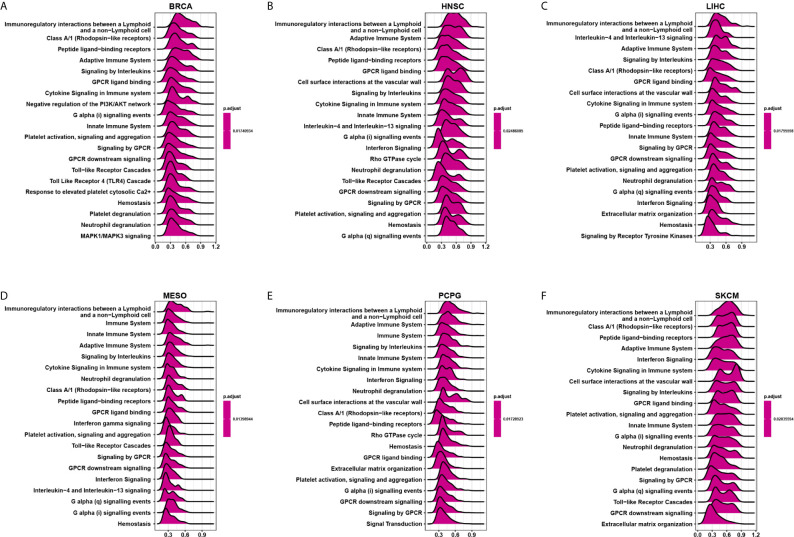
GSEA of CD161 in pan-cancer. **(A–F)**, TOP20 GSEA terms in indicated tumor types.

We evaluated the pathway through which CD161 may involve using GSEA in 33 tumour types from TCGA. The results suggested that CD161 was significantly associated with immune-related pathways, especially for immunoregulatory interactions between lymphoid and non-lymphoid cell pathways, such as in BRCA, HNSC, LIHC, MESO, PCPG, and SKCM (**Figures 6A–F**). These results suggest that CD161 is strongly associated with regulating the tumour immune microenvironment and ligand-receptor interactions between lymphoid and malignant tumour cells.

### Immune Cell Infiltration Analyses

**Figure 7:**
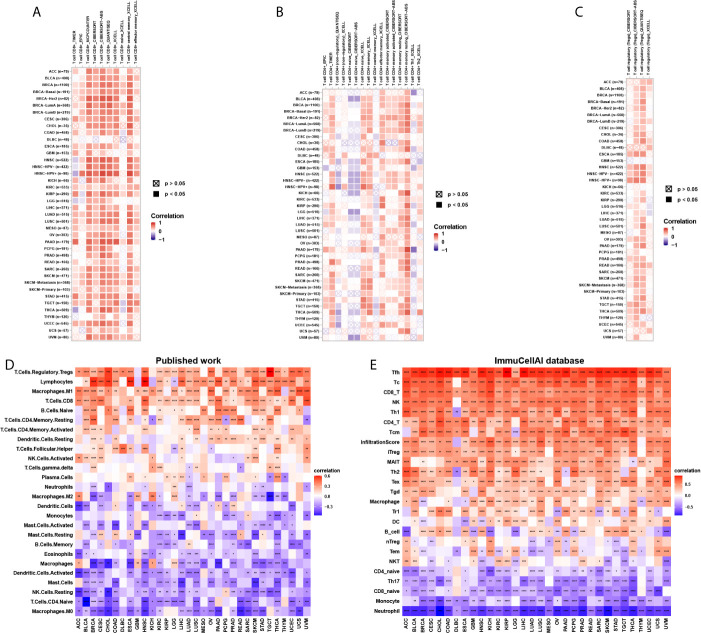
Immune cell infiltration analysis. **(A)**, The correlation between CD161 and infiltration level of CD8+ T cells using TIMER2 database. **(B)**, The correlation between CD161 and infiltration level of CD4+ T cells using TIMER2 database. **(C)**, The correlation between CD161 and infiltration level of Tregs using TIMER2 database. **(D)** The correlation between CD161 and infiltration level of indicated immune cells using data from published work. **(E)** The correlation between CD161 and infiltration level of indicated immune cells using data from ImmuCellAI database. *p < 0.05; **p < 0.01; ***p < 0.001 and ****p < 0.0001.

To explore the relationship between CD161 expression and immune cell infiltration, we conducted correlation analyses using immune cell infiltration data from three different sources. The results of the TIMER2 database revealed that CD161 was positively correlated with the infiltration levels of CD8^+^ T cells, CD4^+^ T cells, and regulatory T cells (Tregs) and negatively correlated with that of naive CD4^+^ T cells in TCGA pan-cancer (**Figures 7A–C**). Correlation analyses using data from published work, which evaluated the 26 immune cells using “CIBERSOFT” method (11), showed that CD161 was positively correlated with the infiltration levels of Tregs, CD8^+^ T cells, and M1-like macrophages, whereas it was negatively correlated with those of M2-like macrophages and naïve CD4^+^ T cells (**Figure 7D**). Correlation analyses using data from the ImmuCellAI database showed that CD161 was positively correlated with the infiltration levels of CD8^+^ T cells, CD4^+^ T cells, natural killer cells, and Tregs and negatively correlated with those of naive CD4^+^ T cells and naïve CD8^+^ T cells (**Figure 7E**). Analysis of immune cell infiltration data from the three different sources was consistent. These results indicate that CD161 may contribute to increased T cell infiltration, which may explain its protective role in most tumour types.

### T Cell Exhaustion Analysis

**Figure 8:**
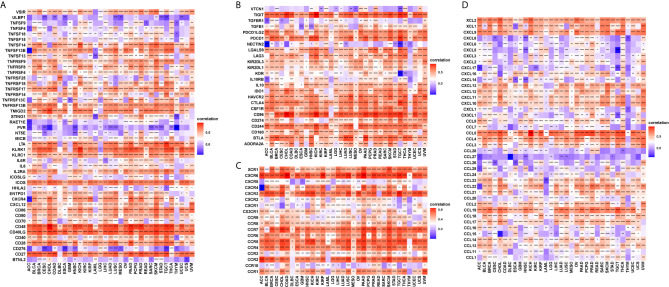
The correlation between CD161 and immunoregulation-related genes. **(A)**, The heatmap represents the correlation between CD161 expression and immune activating genes. **(B)**, The heatmap represents the correlation between CD161 expression and immunosuppressive status related genes. **(C)**, The heatmap represents the correlation between CD161 expression and chemokine receptor genes. **(D)**, The heatmap represents the correlation between CD161 expression and chemokine genes. *p < 0.05; **p < 0.01; ***p < 0.001 and ****p < 0.0001.

T cell exhaustion refers to the loss of T cell functions in patients with common chronic infections and cancer. Patients with tumours often possess a large number of T cells, but most are exhausted. We determined the relationship between CD161 and marker genes of exhausted T cells, immune activating genes, immunosuppressive genes, chemokines, and chemokine receptors. The results revealed that CD161 was positively correlated with marker genes of exhausted T cells, immune activating genes, and immunosuppressive genes in pan-cancer, such as TIGIT, PDCD1, LAG3, CTLA4, STING1, CD96, and IDO1 (**Figures 8A, B**). Immune checkpoints were also closely correlated with CD161 expression in TCGA pan-cancer (**Supplementary Figure 3**). In addition, CD161 expression was positively correlated with chemokines and chemokine receptors, such as CCL4 and CCL5 and their receptors CCR4 and CCR5 (**Figures 8C, D**).

## Discussion

In recent years, immune checkpoint blockade therapy, which changed the landscape of cancer treatment, has become one of the most important immunotherapies in treating cancer (13). Immune checkpoint blocking therapy releases a block in the immune system and induces a lasting anti-cancer response (14–17). Immune checkpoints mainly include PD-1, PD-L1, and CTLA4. CD161, encoded by *KLRB1*, is newly reported as a candidate inhibitor of tumour-infiltrating T cells. Antibody-mediated CD161 blockade enhances T cell-mediated killing of cancer cells *in vitro* and *in vivo* in a few tumour types (5, 6). In this study, we evaluated the role of CD161 using TCGA pan-cancer.

We first assessed the expression and prognostic significance of CD161 in pan-cancer and found that its expression was high in 14 tumours, including ACC, CESC, ESCA, GBM, KIRC, KIRP, LAML, LGG, OV, PAAD, READ, SKCM, STAD, and TGCT. In comparison, low CD161 expression was observed in four tumours, including BLCA, HNSC, LUAD, and LUSC. For the protein information of CD161, we found that the protein level of CD161 was highest in testis cancer, while lowest in lymphoma. To be a potential immune checkpoint, location on cell membrane is necessary. We explored the subcellular location of CD161 and observed that the protein location of CD161 was mainly on the plasma membrane. In addition, we constructed the PPI network and found that CD161 was closely associated with CLEC2D, CLEC2A, GZMA, GZMB, GZMK, KLRC4-KLRK1, CD2, CMKBR6, RORC, and GPR29 proteins.

The Kaplan-Meier OS analysis indicated that CD161 is a protective factor for patients with ACC, BRCA, CESC, CHOL, HNSC, LIHC, MESO, PRAD, PCPG, SARC, SKCM, and THCA and a risk factor for patients with UVM and LGG. For DSS, the results of univariate Cox regression analysis revealed that CD161 acts as a protective factor for patients with ACC, BRCA, CESC, CHOL, HNSC, LIHC, LUAD, READ, SARC, SKCM, and THCA and a risk factor for patients with ESCA, LGG, and UVM. However, the use of OS as the endpoint might diminish the feasibility of clinical studies, and death from non-cancer causes does not necessarily reflect tumour biology, invasiveness, or response to treatment. In addition, using OS or DSS requires longer follow-up time. Therefore, in many clinical trials, the use of DFI or PFI could more effectively reflect the impact of factors on patients. Considering these reasons, we further performed the univariate Cox regression analysis to assess the association between CD161 and DFI or PFI of tumour patients. The DFI analysis revealed that CD161 acts as a protective factor for patients with BLCA, BRCA, CESC, CHOL, COAD, LGG, LIHC, and UCEC. Meanwhile, the PFI analysis showed that CD161 acts as a protective factor for patients with ACC, CESC, CHOL, HNSC, LIHC, LUAD, MESO, SKCM, and UCEC and a risk factor for patients with GBM. These results indicated that high CD161 expression mainly plays a protective role in most tumour types.

Through GSEA of CD161, we found that it was significantly associated with immune-related pathways, especially immunoregulatory interactions between lymphoid and non-lymphoid cell pathways. This pathway comprises of 135 genes, like those encoding receptors and cell adhesion molecules, which play a key role in modifying the response of cells of a lymphoid origin (such as B-, T- and NK cells) to self and tumor antigens, as well as to pathogenic organisms (18–22). These results suggest that CD161 is strongly associated with regulating the tumour immune microenvironment and ligand-receptor interactions between lymphoid and malignant tumour cells.

Cytotoxic CD8^+^ T cells are killer cells in the T lymphocyte population. They support a large amount of cellular immunity, especially in tumour tissues (23, 24). CD4^+^ T cells play an important role in the activation and memory of cytotoxic CD8^+^ T cells (25). We showed that CD161 expression was positively correlated with CD8^+^ T cells, CD4^+^ T cells, and natural killer cells using three different methods, including using TIMER2 database, immune cell infiltration data from a published article (11), and immune cell infiltration data from ImmuCellAI database. This finding may explain the protective role of CD161 in most tumour types. Tregs exert suppressive effects to help malignant tumour cells evade attack from cytotoxic CD8^+^ T cells (26, 27). We observed that Treg infiltration levels were also positively correlated with CD161, indicating that, although the number of cytotoxic CD8^+^ T cells is large, their function may be limited. Notably, CD161 expression was positively correlated with M1-like macrophages and negatively correlated with M2-like macrophages, suggesting that CD161 may have direct or indirect effects on macrophage polarization.

T cell exhaustion refers to the loss of T cell functions in patients with common chronic infections and cancer. Patients with cancer normally possess a large number of T cells, but most of them are exhausted (28). We found that CD161 was positively correlated with marker genes of exhausted T cells, immune activating genes, and immunosuppressive genes in pan-cancer such as TIGIT, PDCD1, LAG3, CTLA4, STING1, CD96, and IDO1. These results confirmed the role of CD161 as an immune checkpoint. Our study has several limitations. The key point is that more *in vivo* experiments should be done to test the antitumor activity by targeting CD161 and additional clinical trials should to be performed to validate the immune checkpoint role of CD161.

In conclusion, we conducted a comprehensive assessment of CD161, revealing its potential role as an indicator of patient prognosis and its immunoregulation effect. As a potential new immune checkpoint, CD161 may be a target for tumour immune therapy.

## Data Availability Statement

The original contributions presented in the study are included in the article/**Supplementary Material**. Further inquiries can be directed to the corresponding authors.

## Author Contributions

LL, DW, CL, and XZ designed the study. CL and XZ performed the data analysis. CL, YC, and HZ wrote the manuscript and helped with the validation. JD helped the revision. All authors contributed to the article and approved the submitted version.

## Funding

This work was supported by the National Nature Science Foundation of China (grant numbers 81773008, 81672756, 81872399, 81972897), the Guangdong Province Universities and Colleges Pearl River Scholar Funded Scheme (2015), the Natural Science Foundation of Guangdong Province (grant number 2017A030311023), the Local Innovative and Research Teams Project of Guangdong Pearl River Talents Program:2017BT01S131 and the Guangzhou Technology Project (grant number 201804010044), National Key R&D Program of China (Grant Nos. 2020YFC2006400), Key-Area Research and Development Program of Guangdong Province (2019B020227004).

## Conflict of Interest

The authors declare that the research was conducted in the absence of any commercial or financial relationships that could be construed as a potential conflict of interest.

## Abbreviations

ACC, Acute Myeloid Leukemia; BLCA, Bladder Urothelial Carcinoma; BRCA, Breast invasive carcinoma; CESC, Cervical squamous cell carcinoma and endocervical adenocarcinoma; CHOL, Cholangiocarcinoma; COAD, Colon adenocarcinoma; ESCA, Esophageal carcinoma; GBM, Glioblastoma multiforme; HNSC, Head and Neck squamous cell carcinoma; KICH, Kidney Chromophobe; KIRC, Kidney renal clear cell carcinoma; KIRP, Kidney renal papillary cell carcinoma; LAML, Acute Myeloid Leukemia; LIHC, Liver hepatocellular carcinoma; LGG, Lower Grade Glioma; LUAD, Lung adenocarcinoma; LUSC, Lung squamous cell carcinoma; MESO, Mesothelioma; OV, Ovarian serous cystadenocarcinoma; PAAD, Pancreatic adenocarcinoma; PCPG, Pheochromocytoma and Paraganglioma; PRAD, Prostate adenocarcinoma; READ, Rectum adenocarcinoma; SARC, Sarcoma; SKCM, Skin Cutaneous Melanoma; STAD, Stomach adenocarcinoma; TGCT, Testicular Germ Cell Tumor; THYM, Thymoma; THCA, Thyroid carcinoma; UCS, Uterine Carcinosarcoma; UCEC, Uterine Corpus Endometrial Carcinoma; UVM, Uveal Melanoma.
