# Supplementary figures and images for: A Pan-Cancer Analysis of CD161, a Potential New Immune Checkpoint
Source: Front Immunol. 2021 Jul 9;12:688215. doi: 10.3389/fimmu.2021.688215 (PMC8299557; doi:10.3389/fimmu.2021.688215)

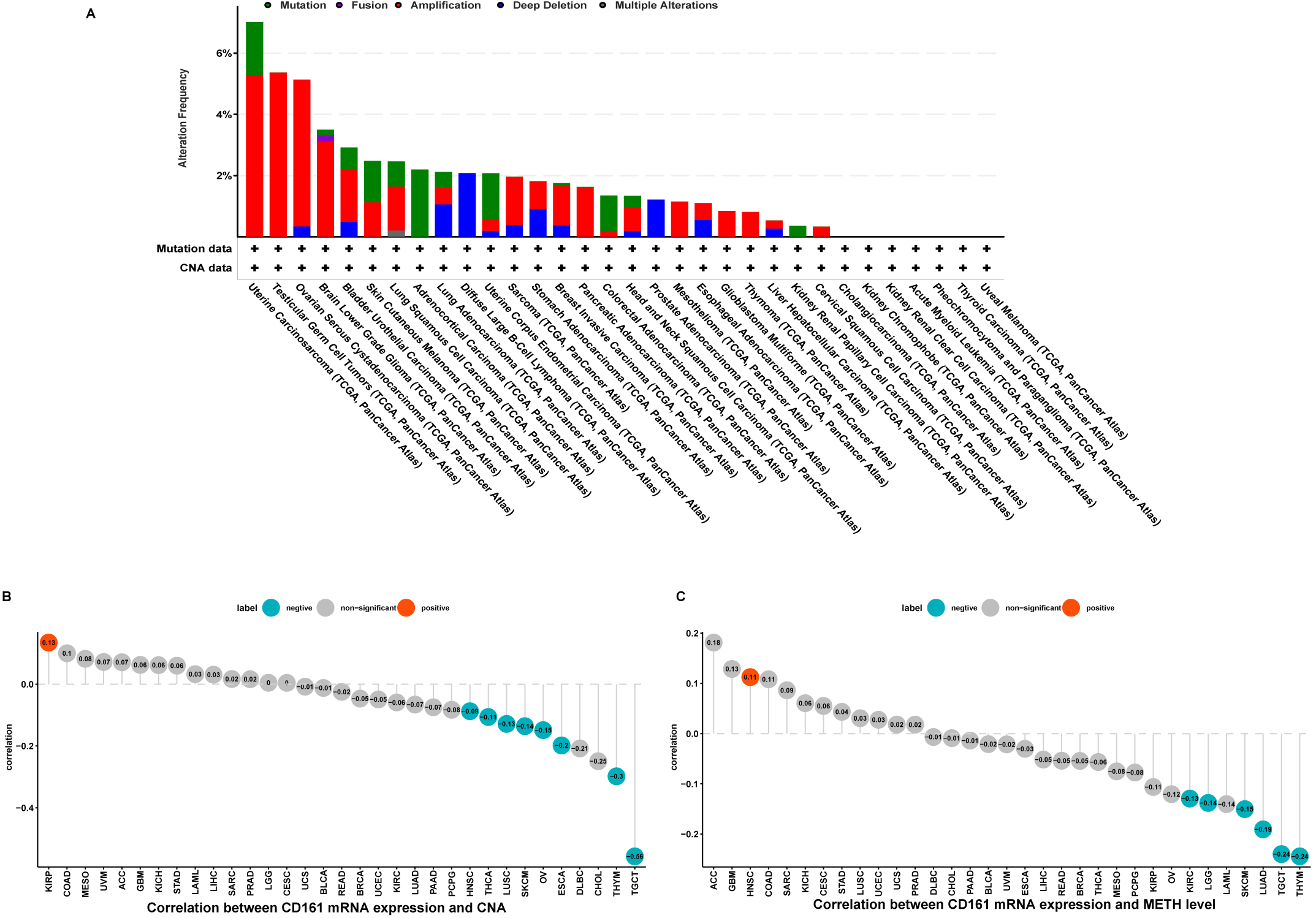

Supplement: Supplementary file 1 [file Image_1.tif]

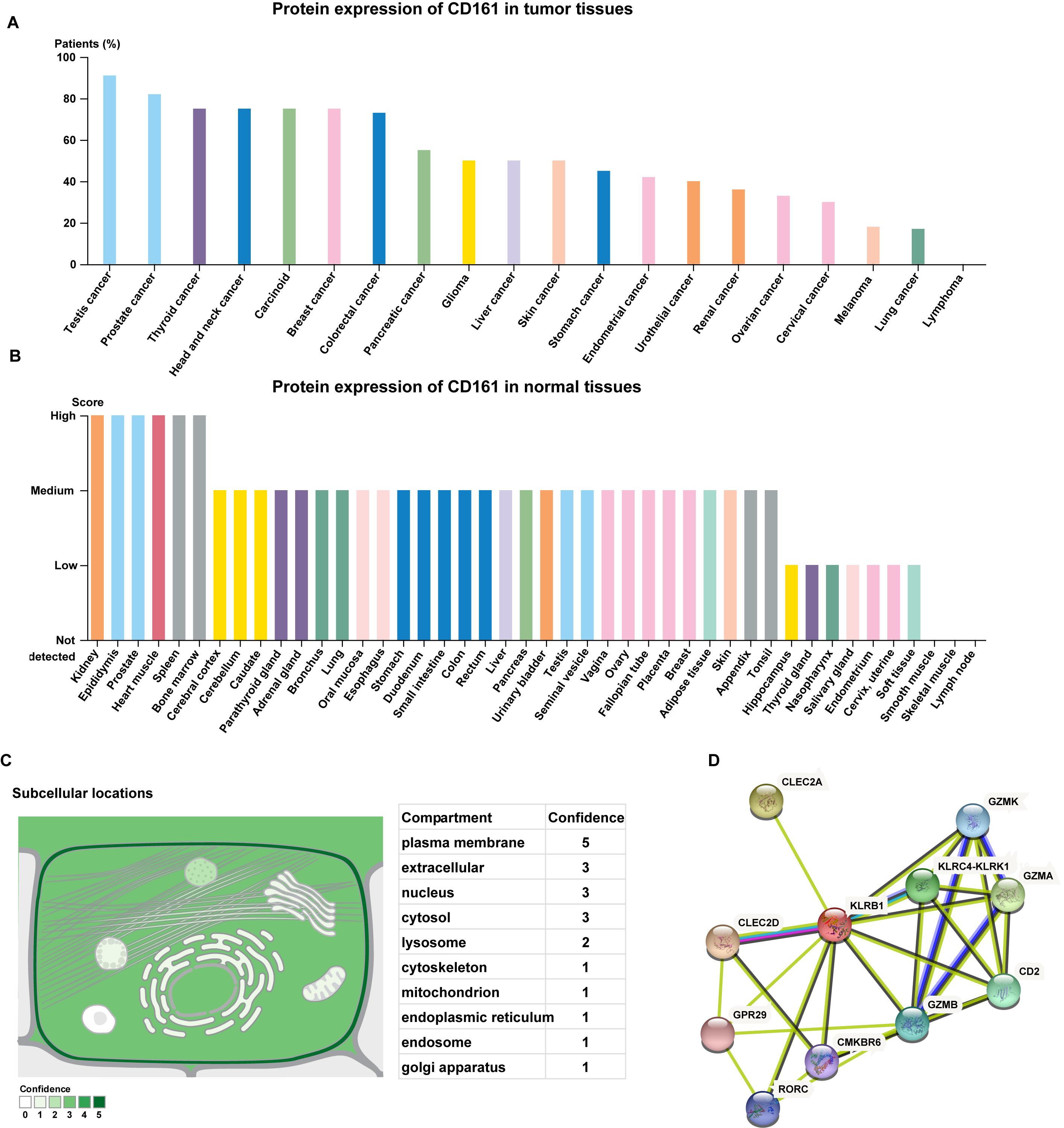

Supplement: Supplementary file 2 [file Image_2.tif]

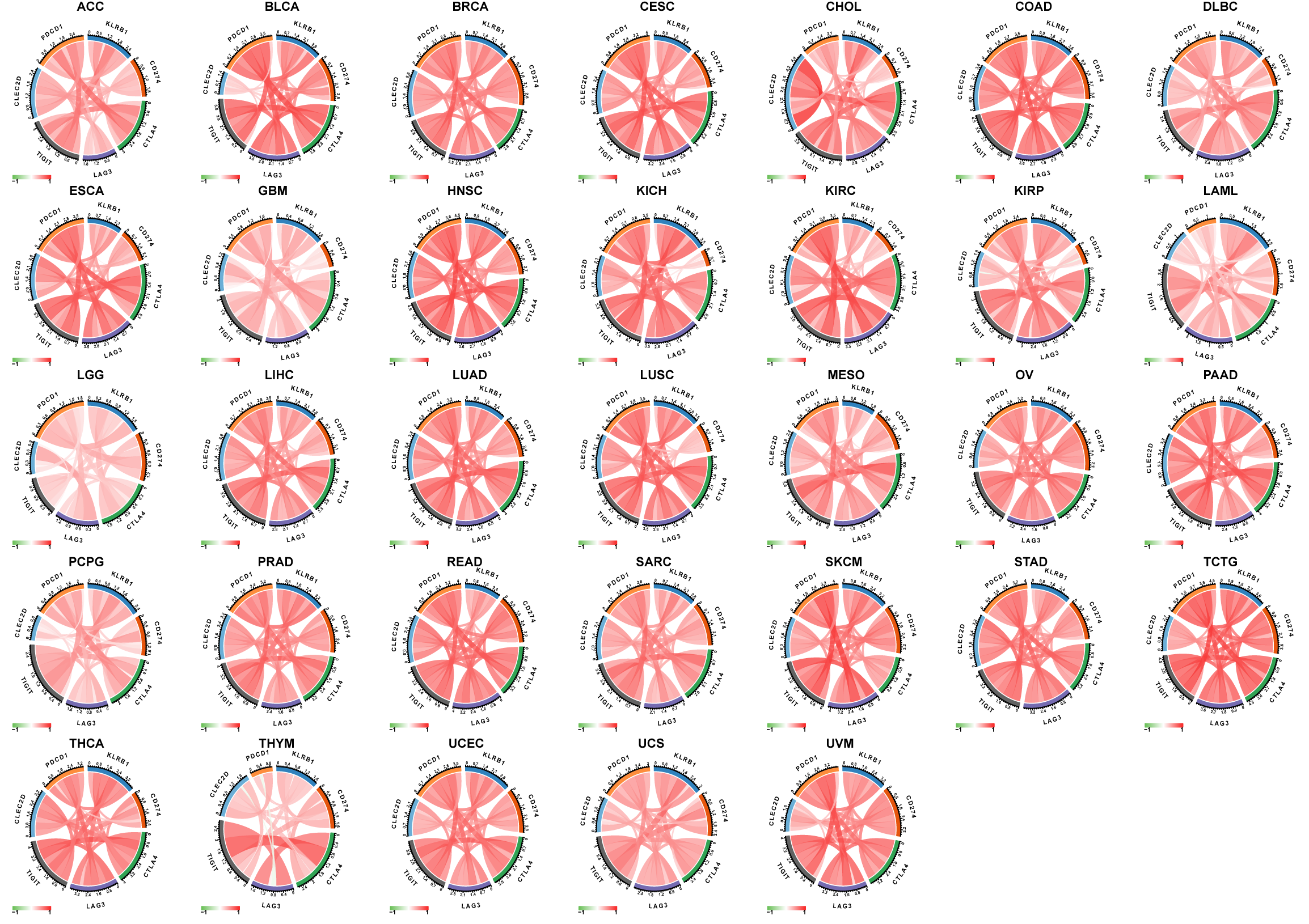

Supplement: Supplementary file 3 [file Image_3.tif]
